# Isolated and repeated stroke-like episodes in a middle-aged man with a mitochondrial ND3 T10158C mutation: a case report

**DOI:** 10.1186/s12883-017-1001-4

**Published:** 2017-12-13

**Authors:** Satomi Mezuki, Kenji Fukuda, Tomonaga Matsushita, Yoshihisa Fukushima, Ryu Matsuo, Yu-ichi Goto, Takehiro Yasukawa, Takeshi Uchiumi, Dongchon Kang, Takanari Kitazono, Tetsuro Ago

**Affiliations:** 1 Stroke Center, St. Mary’s Hospital, 422 Tsubukuhonmachi, Kurume, 830-8543 Japan; 2 Department of Medicine and Clinical Science, Graduate School of Medical Sciences, Kyushu University, 3-1-1 Maidashi, Higashi-ku, Fukuoka, 812-8582 Japan; 3 Department of Mental Retardation and Birth Defect Research, National Institute of Neuroscience, NCNP, 4-1-1 Ogawa-Higashi, Kodaira, Tokyo 187-8551 Japan; 4 Department of Pathological Sciences, Graduate School of Medical Sciences, Kyushu University, 3-1-1 Maidashi, Higashi-ku, Fukuoka, 812-8582 Japan

**Keywords:** Cognitive impairment, MELAS, Mitochondrial ND3 gene, T10158C, Sporadic

## Abstract

**Background:**

Mitochondrial myopathy, encephalopathy, lactic acidosis and stroke-like episodes (MELAS) syndrome, is the most common phenotype of mitochondrial disease. It often develops in childhood or adolescence, usually before the age of 40, in a maternally-inherited manner. Mutations in mitochondrial DNA (mtDNA) are frequently responsible for MELAS.

**Case presentation:**

A 55-year-old man, who had no family or past history of mitochondrial disorders, suddenly developed bilateral visual field constriction and repeated stroke-like episodes. He ultimately presented with cortical blindness, recurrent epilepsy and severe cognitive impairment approximately 6 months after the first episode. Genetic analysis of biopsied biceps brachii muscle, but not of peripheral white blood cells, revealed a T10158C mutation in the mtDNA-encoded gene of NADH dehydrogenase subunit 3 (*ND3*), which has previously been thought to be associated with severe or fatal mitochondrial disorders that develop during the neonatal period or in infancy.

**Conclusion:**

A T10158C mutation in the ND3 gene can cause atypical adult-onset stroke-like episodes in a sporadic manner.

## Background

It is well established that point mutations and deletions of mitochondrial DNA (mtDNA) cause mitochondrial disorders. While mtDNA point mutations are usually maternally inherited, deletions usually occur sporadically. Mitochondrial myopathy, encephalopathy, lactic acidosis and stroke-like episodes (MELAS), is the most common phenotype of mitochondrial disease. The stroke-like episodes often develop in childhood or adolescence, and usually before the age of 40 [1]. Among the reported mutations in mtDNA, an A to G mutation at nucleotide position 3243 (A3243G) in the mitochondrially encoded tRNA leucine 1 (MT-TL1) gene, which encodes a transfer RNA for leucine, has been found in more than 80% of MELAS patients [2]. The A3243G mutation ultimately leads to multiple defects of the mitochondrial respiratory chain (MRC) [3]. Complex I deficiency accounts for many cases of MRC defects and results in a wide range of mitochondrial diseases [4]. It has been thought that mutations in Complex I subunits often manifest a severe or fatal mitochondrial disorder from the neonatal period to infancy, known as Leigh syndrome [3].

Here we describe the case of a 55-year-old man who sporadically and suddenly developed repeated stroke-like episodes, ultimately presenting with cortical blindness, recurrent epilepsy and severe cognitive impairment. We identified a T10158C mutation in the NADH dehydrogenase subunit 3 (*ND3*) gene in this patient. We discuss the significance of this mutation in the development of sporadic adult-onset stroke-like encephalopathy.

## Case presentation

**Fig. 1:**
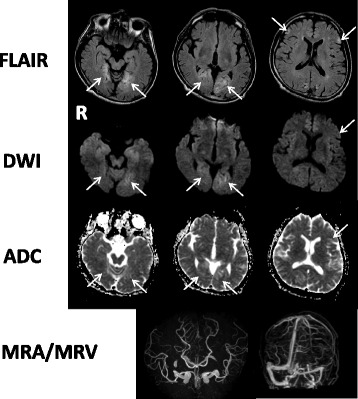
Fluid-attenuated inversion recovery (FLAIR) images and diffusion-weighted images (DWI) show multiple high-intensity signals (arrows) in the cortical and subcortical areas of the bilateral occipital and the mesial temporal lobes, and the left frontal lobe. Apparent diffusion coefficient (ADC) images show normal- to slightly low-intensity signals in the areas with the abnormal signals shown by FLAIR and DWI (arrows). Magnetic resonance angiography and venography (MRA and MRV) show no abnormalities

**Fig. 2:**
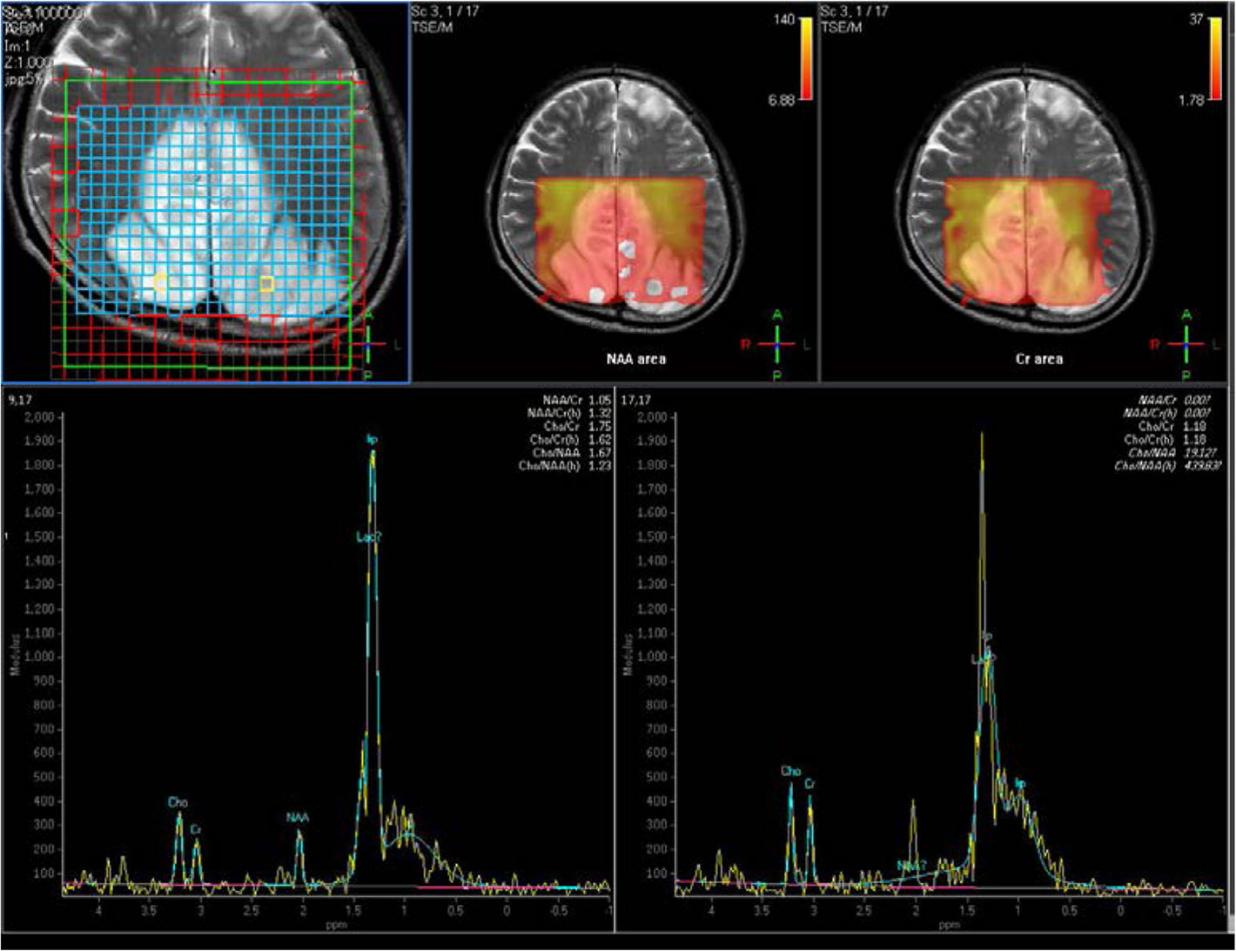
Magnetic resonance spectroscopy (MRS) reveals markedly elevated lactate (Lac) concentrations in the regions of interest in the high-intensity area of the bilateral occipital lesion (arrows). It also reveals decreased N-acetyl acetate (NAA) and slightly elevated choline (Cho) in these areas

**Fig. 3:**
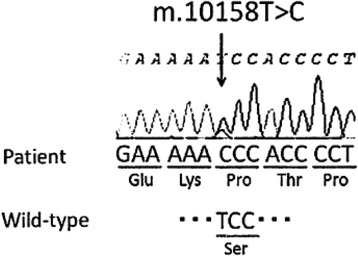
Identification of the T10158C mutation in the ND3 gene by sequencing mitochondrial DNA obtained from the right biceps brachii muscle

**Fig. 4:**
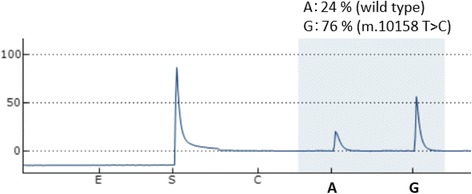
Pyrosequence analysis of mitochondrial DNA extracted from biopsied muscle

A previously healthy 55-year-old Japanese man became aware of flashing lights in his visual field. A month later he visited a doctor with a complaint of sudden visual field constriction. Magnetic resonance images (MRI) revealed abnormal signals in the bilateral occipital lobe, and he was transferred to our hospital. At the time of admission, the height of the patient was 167 cm and his weight was 57 kg. His blood pressure was 158/70 mmHg, and his heart rate was 72 beats per minute and regular. His body temperature was 36.8 °C. Although his vision was slightly misty, he was well orientated and his higher brain function was normal. He had afferent tunnel vision, but no other obvious neurological deficits. Blood tests, including serum autoantibodies and soluble IL-2 receptor antibody, indicated no apparent abnormalities except that the ratio of lactate (47.5 mg/dl) to pyruvate (2.09 mg/dl) was significantly increased (L/P ratio 22.7). Cerebrospinal fluid (CSF) examination showed a normal cell count (1/μl) and a slightly elevated protein concentration (55 mg/dl). His CSF L/P ratio was also increased (lactate 27.6 mg/dl, pyruvate 1.30 mg/dl and L/P ratio 21.2). Levels of adenosine deaminase and 14–3-3 protein in CSF were within normal limits. Both fluid-attenuated inversion recovery (FLAIR) and diffusion-weighted images (DWI) revealed multiple high-intensity signals in the cortical and subcortical areas of the bilateral occipital lobe, the mesial temporal lobes, and the left frontal lobe (Fig. 1). Apparent diffusion coefficient (ADC) images showed normal- to slightly low-intensity signals in the areas with the abnormal signals shown by FLAIR and DWI (Fig. 1). Magnetic resonance angiography and venography (MRA and MRV) showed no abnormalities (Fig. 1). Furthermore, magnetic resonance spectroscopy (MRS) demonstrated elevated lactate concentrations in the bilateral occipital lesion (Fig. 2, arrows). Electroencephalography showed periodic slow waves and sharp waves bilaterally, but no periodic synchronous discharge was detected. No abnormalities were revealed by ophthalmological examinations, audiography, echocardiography, electromyography, or glucose tolerance tests. Histopathological studies of biopsied right biceps brachii muscle showed no mitochondrial myopathic features, including ragged-red fibers or intense succinate dehydrogenase activity. However, genetic analysis of the biopsied muscle by complete sequencing of mtDNA revealed a T10158C missense mutation in the *ND3* gene (Fig. 3). Pyrosequence analysis, a method used to quantify heteroplasmy [5], showed that 76% of the mtDNA in the biopsied muscle of the patient had the mutation (Fig. 4). The mutation was not found in DNA obtained from his peripheral white blood cells.

**Fig. 5:**
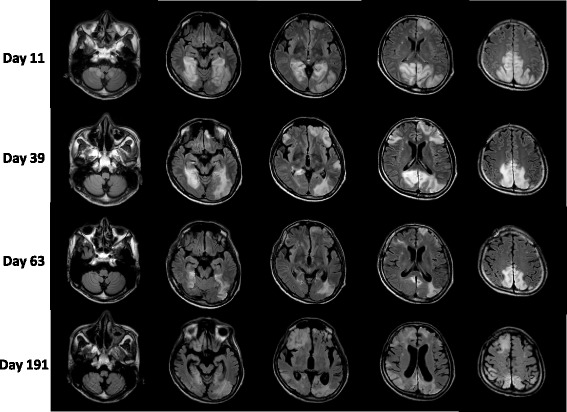
FLAIR images at days 11, 39, 63 and 191 show high intensity signals appearing recurrently in various cortical and subcortical areas, mainly in the bilateral occipital lobe, and that atrophic changes develop progressively over approximately 6 months

After the first episode, he had repeated stroke-like episodes accompanied by abnormal MRI signals in various areas, mainly in the occipital lobes. Within 6 months he manifested cortical blindness, recurrent epilepsy and severe cognitive impairment (Fig. 5).

## Discussion and conclusions

Here we described a middle-aged man who suddenly developed repeated stroke-like episodes and eventually presented with cortical blindness, recurrent epilepsy and severe cognitive impairment within about 6 months. We considered that ischemic stroke, sinus thrombosis, posterior reversible encephalopathy syndrome, central nervous system (CNS) vasculitis, encephalopathies, malignant lymphoma, and Creutzfeldt-Jacob disease could be excluded based on laboratory examinations, including blood and CSF, physiological examinations, MR imaging, and clinical course. Instead, we identified a significant increase in the ratio of lactate to pyruvate in both his blood and CSF and a heteroplasmic T10158C mutation in the *ND3* gene, encoded on mtDNA, in his right biceps brachii muscle but not in peripheral white blood cells. We therefore suggest that a possible diagnosis for the patient could be isolated mitochondrial encephalopathy associated with the mutation.

The gene product of ND3, NADH dehydrogenase subunit 3, is a structural component of the multimeric enzyme Complex I of the MRC, and is involved in the active/de-active enzymatic transition of Complex I [6]. Because Complex I drives ATP generation in the mitochondria, any genetic abnormalities of its components could directly cause severe impairment of energy generation and subsequent cellular or organ failure, particularly in the CNS. Currently, three *ND3* mutations that can lead to MRC defects are known: T10158C [7–9], T10191C [10–12] and G10197A [4, 13]. Although it has been reported that most of the cases harboring these mutations manifested mitochondrial encephalopathy during the neonatal period or in infancy [7, 12], Mukai et al. recently reported two adult cases with the T10158C mutation that developed repeated stroke-like episodes [8, 9] and are very similar to the present case. Their cases and ours had common clinical characteristics: 1) the patients developed repeated stroke-like episodes later than middle-age, 2) the CNS was affected in an isolated manner and 3) they had no recognized family history of mitochondrial disorders.

Adult-onset stroke-like episodes are a common feature of MELAS syndrome where more than 80% patients harbor the A3243G mutation [2]. In contrast to MELAS patients with the A3243G mutation, it is generally understood that patients with mutations in Complex I are likely to manifest severe and fatal mitochondrial disorders, such as Leigh syndrome, in the neonatal to infant period [14]. However, the fact that these three cases harbor the T10158C mutation in the ND3 subunit of Complex I, causing late-onset stroke-like episodes similar to MELAS, highlights the complexity of the relationship between the causative mutation and clinical manifestation.

Various mtDNA mutations can cause either multiple or single organ disorders due to their heteroplasmic characteristics. These three adult-onset cases with the T10158C mutation manifested isolated encephalopathy, presenting with stroke-like episodes, but not myopathy or other organ dysfunctions. This is despite the fact that the mutation was identified in biopsied muscles with an approximately 80% mutation load [8]. This may be attributable to “threshold effects”: it is thought that threshold values of mutational load presenting phenotypic changes may be around 60% for mtDNA deletions, while around 90% for mtDNA point mutations [15]. We therefore speculate that the mutational load in the affected CNS lesions in the present case may be over 90%. Alternatively, the mutation may disrupt the functions of the CNS more easily than those of other organs, such as muscles, even with a similar mutational load of around 80%. We would need to perform a brain biopsy and genetic analysis to determine this.

Pathogenic mtDNA mutations can be maternally inherited or occur sporadically. Although it is thought that point mutations are usually inherited maternally [1], there were no recognized family histories of mitochondrial disorders, with either infantile- [7] or adult- [8, 9] onset, in the reported cases with the T10158C mutation. This indicates that the T10158C mutation may occur de novo in the germline cells of the patients’ mothers and may attain high-level heteroplasmy, presenting encephalopathy very rapidly in some cases [7], or slowly over 40 years in others. We infer the presence of additional unknown factors that accelerate or slow the dysfunction or accumulation of mtDNA with the T10158C mutation [16].

Our study supports the hypothesis that the T10158C mutation in the mitochondrial *ND3* gene can sporadically cause isolated and repeated stroke-like episodes, with an onset later than middle-age [8, 9].

## Abbreviations

ADC: Apparent diffusion coefficient
CNS: Central nervous system
CSF: Cerebrospinal fluid
DWI: Diffusion-weighted images
MELAS: Mitochondrial myopathy, encephalopathy, lactic acidosis and stroke-like episodes
MRC: Mitochondrial respiratory chain
MRI: Magnetic resonance images
MRS: Magnetic resonance spectroscopy
